# Neural temporal dynamics of stress in comorbid major depressive disorder and social anxiety disorder

**DOI:** 10.1186/2045-5380-2-11

**Published:** 2012-06-22

**Authors:** Christian E Waugh, J Paul Hamilton, Michael C Chen, Jutta Joormann, Ian H Gotlib

**Affiliations:** 1 Department of Psychology, Wake Forest University, P.O. Box 7778, Winston Salem, NC, 27109, USA; 2 Department of Psychology, Stanford University, Stanford, CA, USA; 3 Department of Psychology, University of Miami, Coral Gables, FL, USA

**Keywords:** Depression, Anxiety, Comorbidity, FMRI, Stress, MPFC

## Abstract

**Background:**

Despite advances in neurobiological research on Major Depressive Disorder and Social Anxiety Disorder, little is known about the neural functioning of individuals with comorbid depression/social anxiety. We examined the timing of neural responses to social stress in individuals with major depression and/or social anxiety. We hypothesized that having social anxiety would be associated with earlier responses to stress, having major depression would be associated with sustained responses to stress, and that comorbid participants would exhibit both of these response patterns.

**Methods:**

Participants were females diagnosed with pure depression (n = 12), pure social anxiety (n = 16), comorbid depression/social anxiety (n = 17), or as never having had any Axis-I disorder (control; n = 17). Blood oxygenation-level dependent activity (BOLD) was assessed with functional magnetic resonance imaging (fMRI). To induce social stress, participants prepared a speech that was ostensibly to be evaluated by a third party.

**Results:**

Whereas being diagnosed with depression was associated with a resurgence of activation in the medial frontal cortex late in the stressor, having social anxiety was associated with a vigilance-avoidance activation pattern in the occipital cortex and insula. Comorbid participants exhibited activation patterns that generally overlapped with the non-comorbid groups, with the exception of an intermediate level of activation, between the level of activation of the pure depression and social anxiety groups, in the middle and posterior cingulate cortex.

**Conclusions:**

These findings advance our understanding of the neural underpinnings of major depression and social anxiety, and of their comorbidity. Future research should elucidate more precisely the behavioral correlates of these patterns of brain activation.

## Background

Although investigators have examined the psychological and neurobiological mechanisms that underlie Major Depressive Disorder (MDD) and Social Anxiety Disorder (SAD), most researchers have assessed these disorders separately. Over 50 percent of people diagnosed with a psychological disorder, however, have additional comorbid disorders [1]. Depression is likely to co-occur with forms of anxiety and, in particular, with social anxiety [2]. Indeed, individuals with comorbid depression/social anxiety (MDD/SAD) have poorer behavioral (e.g. strengthened negative cognitive biases) [3] and life (e.g. likelihood of suicide attempt) [4] outcomes than do people with either diagnosis alone.

These deleterious consequences of comorbid depression and social anxiety underscore the importance of elucidating the neural mechanisms that underlie this comorbidity. After decades of research, investigators now have formulated neural models of depression [5] and of social anxiety (e.g.,) [6] based on patterns of neural activation that have been found in these disorders. For example, in response to emotional stimuli, depressed participants exhibit abnormal increases in amygdala [7] and subgenual anterior cingulate cortex activity, [8] and an abnormal lack of dorsolateral prefrontal cortex activity [9]. In response to emotional stimuli, participants with social anxiety exhibit hyperactivity in the amygdala (similar to depressed participants), [6] as well as hyperactivity in the rostral anterior cingulate cortex [10] and insula [11]. These neural models, however, are based on findings of studies conducted with pure, non-comorbid forms of these disorders. We know much less about the neural functioning of individuals with comorbid depression/social anxiety – whether comorbid individuals exhibit neural responses that are more similar to the responses of people with one disorder or the other, an additive combination of the disorder-specific responses, or an intermediate blend of the disorder-specific responses.

Recent studies highlight the utility of examining the neural functioning of individuals with comorbid disorders. For example, Andreescu et al. [12] found that older participants with diagnosed major depression and high scores on a self-report measure of anxiety exhibited greater activation than did depressed participants with low anxiety scores in the dorsal anterior cingulate cortex in a cognitive interference task. More recently, Etkin and Schatzberg [13] examined the neural functioning of patients diagnosed with depression and/or Generalized Anxiety Disorder while they performed an emotional conflict task and found that, whereas activation deficits in the ventral cingulate and amygdala were shared by all patient groups, activation in the lateral prefrontal cortex was unique to the non-comorbid depression group. These findings demonstrate that examining individuals with comorbid disorders can distinguish patterns of neural functioning that are common to both disorders from those that are unique to each disorder. In the present study, we both extend these findings of studies assessing other comorbid disorders to examine the neural mechanisms that underlie the comorbidity of depression and social anxiety, and advance research on neural functioning in comorbid disorders by examining the temporal dynamics of whole-brain responses to stress.

Given the importance of stress in the etiology and maintenance of both depression [14] and anxiety, [15] it is likely that examining the neural correlates of the stress response will prove critical in understanding neural aspects of comorbidity. For example, Young and colleagues found that individuals with comorbid depression and anxiety (including, but not limited to social anxiety) exhibited greater hypothalamic-pituitary-adrenal (HPA) axis responses to social stress than did individuals diagnosed with either depression or anxiety alone [16]. One potentially illustrative difference between anxious and depressed persons is the timing of their responses to stress. Anxious people are characterized by a vigilance-avoidance attentional bias in which they both engage with and disengage from threat-related stimuli relatively quickly [17-19] (although there is mixed evidence for the avoidance from threat-related stimuli in social anxiety) [20], which may be one mechanism underlying their enhanced responses during the anticipation of stressors [11]. In contrast, depressed individuals are characterized by high levels of rumination about past negative emotional stimuli, [21,22] which may underlie their more sustained responses to stress [23].

Recently, investigators have used social evaluative threat tasks to assess temporal aspects of the neural response to stress in humans. Social evaluative threat tasks induce robust changes in both hypothalamic-pituitary-adrenal axis activity [24] and peripheral nervous system activity [25]. Investigators have demonstrated that the association between social threat and these stress-induced physiological responses is mediated by the rostral medial frontal cortex [26]. This medial frontal region exhibits activation early in the stressor as well as sustained activation throughout the duration of the stressor [26,27]. Although this may represent a normal response profile to stress, the finding that the medial frontal cortex is involved in both the initial generation and subsequent maintenance of the stress response highlights this region as a possible site for activation patterns hypothesized to characterize both social anxiety and depression. Whereas the hyper-vigilance to threat in socially anxious people may affect medial frontal cortex activation early in the stressor, the sustained responses to threat in depressed people may influence medial frontal cortex activation late in the stressor.

In the current study, we examined the magnitude and timing of neural responses to social stress in participants diagnosed as having pure depression, pure social anxiety, comorbid depression/social anxiety, or as never having had any Axis-I disorder. We hypothesized that relative to participants without social anxiety, participants diagnosed with social anxiety will exhibit greater changes in medial frontal cortex activation from baseline to early in the stressor (i.e., during instructions or early speech preparation) and less activation late in the stressor (i.e., during late speech preparation or recovery). Alternatively, relative to participants without depression, participants diagnosed with depression will exhibit greater responses in the medial frontal cortex later in the stressor. Finally, it is not clear whether the effects of comorbidity are additive or interactive. We hypothesized that if comorbidity is the additive combination of depression and social anxiety, then comorbid participants should exhibit medial frontal cortex responses both early and late in the stressor.

## Methods

### Participants

Participants were recruited from local psychiatric outpatient clinics and through website postings. Sixty-four female individuals participated in this study: 16 participants diagnosed with social anxiety disorder; 14 participants diagnosed with major depressive disorder; 17 participants diagnosed with comorbid major depressive disorder and social anxiety disorder; and 17 never-disordered control participants. Diagnostic evaluations were based on DSM-IV-TR criteria using the Structured Clinical Interview for DSM-IV-TR Axis I (SCID-I) [28] administered by trained interviewers. Participants in the comorbid group met diagnostic criteria for both current social anxiety disorder and current major depressive disorder, but did not meet current or lifetime criteria for any other Axis-I disorder. Participants in the two non-comorbid clinical groups met diagnostic criteria for either current social anxiety disorder or major depressive disorder, but did not meet criteria for lifetime comorbidity of anxiety and depression or for any other current or lifetime Axis-I disorder. Finally, controls did not meet criteria for current or past Axis-I psychopathology. Inter-rater reliability kappas ranged from .92 to 1.0 for the above diagnoses. All participants were between 18 and 55 years of age, had no lifetime history of psychotic ideation and no reported substance abuse within the past six months. All aspects of this study complied with the ethical standards for treatment of human participants from the American Psychiatric Association and were approved by the Institutional Review Board. All participants provided informed consent. Due to equipment issues, data from two participants (both from the non-comorbid major depressive disorder group) were excluded from further analyses.

### Demographic and clinical information

Participants provided demographic and clinical information, including age, education, household income, race, symptoms of social anxiety (Social Phobia Anxiety Index) [29] and depression (Beck Depression Inventory - II), [30] treatment status (Table 1), and current medications (Additional file 1: Table S1).

**Table 1:** Demographic characteristics, clinical characteristics, and self-reported responses to the social evaluative threat task of the diagnosed groups

	**Depression (n = 12)**	**Social Anxiety (n = 17)**	**Comorbid (n = 17)**	**Control (n =16)**	**Interaction of depression and social anxiety**	**Main effect of having depression**	**Main effect of having social anxiety**
G^2^							
Race (%Caucasian)	75%	71%	76%	50%	5.10	2.34	1.84
Current treatment	33%	13%	35%	0%	9.82*	7.22*	.60
F							
Age	39.67 (11.30)	32.82 (12.34)	29.88 (10.92)	33.64 (9.36)	2.40	.28	3.36
Income^ab^	3.70 (2.11)	4.08 (1.44)	2.60 (1.55)	4.00 (1.75)	1.52	3.47	1.15
Education (years)^c^	15.80 (2.69)	16.06 (1.12)	14.92 (2.25)	17.80 (2.73)	.48	6.27*	4.32*
Depression (BDI-II)	26.58 (13.85)	11.65 (8.82)	30.35 (10.83)	2.06 (3.15)	.63	100.85*	13.88*
Social Anxiety (SPAI)	63.31 (29.67)	91.47 (26.11)	98.55 (17.81)	29.79 (28.88)	3.70	8.73*	49.71*
Anti-Depressant Load	1.75 (2.01)	.41 (1.18)	1.12 (2.12)	.00 (.00)	1.74	9.61*	.08
Anti-Anxiety Load	.33 (.65)	.12 (.33)	.18 (.39)	.00 (.00)	1.86	3.80*	.04
Perceived demands	4.78 (1.44)	5.45 (1.18)	5.45 (1.39)	3.74 (1.35)	2.31	2.29	12.09*
Perceived resources	3.94 (1.25)	3.73 (1.71)	2.96 (1.37)	5.04 (1.05)	.21	6.81*	10.39*
Positive affect during speech prep	1.98 (.94)	1.90 (.68)	1.74 (.83)	2.58 (.94)	1.03	3.05	4.40*
Negative affect during speech prep	3.02 (1.21)	3.32 (1.12)	3.45 (1.04)	2.26 (.82)	1.35	2.70	7.63*
Positive affect after speech prep	2.31 (.88)	2.30 (.82)	2.13 (.59)	2.78 (1.03)	.44	2.20	2.30
Negative affect after speech prep	1.22 (.46)	1.33 (.42)	1.53 (.64)	1.29 (.58)	.93	.20	1.60

BDI-II = Beck Depression Inventory; SPAI = Social Phobia and Anxiety Inventory; PA = positive affect; NA = negative affect. Standard deviations are in parentheses. * p < .05.

^a^ Income was measured on an ordinal scale (1 = less than 10 k, 2 = 10-25 k, 3 = 25-50 k, 4 = 50-75 k, 5 = 75-100 k, 6 = more than 100 k).

^b^ N = 52 (Depression, n = 10; Social Anxiety, n = 13; Comorbid, n = 15; Control, n = 14).

^c^ N = 50 (Depression, n = 11; Social Anxiety, n = 16; Comorbid, n = 13; Control, n = 10).

### Social evaluative threat task

In the scanner, participants completed a social evaluative threat task (450 seconds; 7:30) [26]. All instructions were presented visually. For baseline (120 seconds), participants were instructed to relax, to clear their minds of all thoughts and feelings, and to keep their eyes on a fixation cross. In the task instructions period (80 seconds), participants were told that they would have 2 minutes to prepare a 7-minute speech on “Why you are a good friend,” that they would be audiotaped, that others would evaluate their performance, and that there was a possibility that they would not have to give their speech. In the speech preparation period (120 seconds), participants were instructed to ‘Prepare your speech now,’ which remained on the screen for the entire period. In the recovery period (130 seconds), participants were notified that they would not have to give a speech and were instructed to wait for the next task to begin; throughout this period, participants viewed a fixation cross. This speech task has been used successfully in several studies to induce robust affective [25], cardiovascular, [25,31] and neural responses [26].

### fMRI Data Acquisition

BOLD data were acquired with a 3.0 T General Electric Signa MR scanner. Following scout scanning, high order shimming was performed over the whole brain until diminishing returns on image distortion correction were met. Next, BOLD data were acquired with a single channel, whole-head imaging coil from 31 axial slices using a spiral pulse sequence [32] [repetition time = 2000 ms, echo time = 40 ms, flip angle = 70°, field of view = 220 mm, number of frames = 225]. Axial slices had 3.44 mm^2^ in-plane and 4 mm through-plane resolution (with 1 mm between-slice distance). A high resolution structural scan (115 slices, 1 mm^2^ in-plane and 1.5 mm through-plane resolution, echo time = min, flip angle = 15°, field of view = 22 cm) was performed following BOLD scanning runs.

### fMRI Image Preprocessing

Preprocessing was performed with the AFNI imaging analysis suite [33]. BOLD images were slice-time corrected to the fifteenth axial slice and motion corrected to the middle acquisition slice using Fourier interpolation. Data were then spatially smoothed with an 8 mm Gaussian kernel and warped to Talairach template space (voxel size = 3 mm^3^) [34]. Next, multiple regression was used to remove nuisance effects including six head movement parameters (estimated from motion correction procedure), whole-brain global time series, and linear drift. These images were then converted to statistical parametric mapping (SPM) analyze format and gray-matter masked with the participants’ averaged structural image.

### fMRI data analysis

To determine *where* and *when* the four groups differed in their brain activation during the social evaluative threat task, we conducted both standard GLM analyses and change-point analyses [27] on the preprocessed images using a combination of SPM and custom MATLAB (Mathworks, Inc.) scripts. For both analyses, each participant’s data were first temporally smoothed (6 s kernel; according to published specifications) [27] with an exponentially weighted moving average.

### GLM analysis

We first conducted a standard GLM analysis to determine whether this simpler analysis could capture temporal differences in activation patterns as a function of diagnosis. The smoothed time-series data were regressed on activation functions that modeled each task period (i.e. 1 s during the task period of interest and 0 s everywhere else). The task periods of interest included the instructions, early speech preparation, late speech preparation, and recovery periods. The participants’ beta coefficients were then submitted to a multiple regression model. Similar to factorial analyses conducted in previous fMRI studies examining the comorbidity of depression and anxiety [13], we entered into this group-level regression model mean-scaled dummy variables representing a) being diagnosed with depression or not (MDD or MDD/SAD [1] vs. CTL or SAD [−1]); b) being diagnosed with social anxiety or not (SAD or MDD/SAD [1] vs. MDD or CTL [−1]); and c) their interaction (the product of the first two predictors). We also added into our model medication covariates reflecting both antidepressant and anxiolytic/sedative loads as regressors of non-interest (See Additional file 1: Table S1) [35]. The resulting whole-brain t-maps were corrected (threshold = .0005, k = 14) to derive per-voxel corrected p-values < .05 (based on 1000 Monte Carlo simulations) [36].

### Change-point analysis

The primary difference between the GLM analysis and the change-point analysis is that instead of estimating activation for an entire task period, the change-point analysis estimates activation for every time-point after baseline. To do this, we first subtracted activity during the baseline period from activity from the task periods of interest (everything after baseline). Next, we conducted weighted multiple regression (using the same group-level regression model mentioned above) on every time-point in each voxel’s time-series [27]. Similar to the GLM, ‘activation’ still reflects changes from baseline, and ‘activation differences’ still reflects changes in activation between diagnostic groups. Because the change-point analysis conducted hundreds of regressions for each voxel (for each time point), we implemented family-wise error control with a Monte Carlo simulation (5000 iterations) to get corrected p-values across each voxel’s time-series. A change-point in activation was defined as the longest series of consecutive time-points exhibiting suprathreshold activation levels or differences in activation as a function of our group-level regressors (minimum of 3 time-points to capture meaningful changes in activation; corrected p < .05). This created three separate activation maps: 1) a map of the max t within a change-point series, which corresponds roughly to standard GLM contrast maps; 2) a map of the first suprathreshold time-point of the change-point series to determine when these change-points began; and 3) a map of the duration of the change-point series. For ease of presentation, we categorized the change-point onsets as occurring during the instructions (60–100 time-points), early speech preparation (101–130 time-points), late speech preparation (131–160 time-points), or recovery period (161–225 time-points).

Next, to derive maps of the simple main effects of diagnoses that were not qualified by the interaction of the two diagnoses, we masked the main effects max-t activation maps with the interaction map. In addition, to limit diagnosis-related differences in activation to those areas that were specifically responsive to the social evaluative threat task, we masked the main effects and interaction max-t activation maps with the max-t activation map from the group intercept. These steps were not necessary for the GLM analyses because we found no main effects or interaction of diagnoses. These change-point max-t activation maps were then thresholded with the same criteria used for the GLM analyses.

For post-hoc contrasts tests, we conducted simple-slopes analyses [37] using the average of the beta-coefficients across all the suprathreshold time-points for all the voxels in each cluster. For post-hoc correlations, we averaged each participant’s BOLD responses across all the suprathreshold change-point TRs for each voxel in the clusters of interest. We corrected for multiple correlations using Bonferroni correction.

### Self-report responses to the social evaluative threat task

#### Speech appraisals

After participants exited the MRI scanner, they were asked to rate how they would feel “right now if you would have had to give the speech” on scales assessing appraisals of stress [38]. Participants provided responses from 1 (*strongly disagree*) to 7 (*strongly disagree*) on two subscales: one assessing the perceived demands of the social evaluative threat task (e.g. “The speech would have been very demanding;” “The speech would have been very stressful”), and the other assessing the participants’ perceived resources available to engage in the speech (e.g. “I feel that I would have had the abilities to perform well in the speech”). The ratio of perceived demands to perceived resources has been found to underlie feelings of threat [39]. The two subscales exhibited good reliability (demands: α = .86; resources: α = .84).

### Affect

Participants were also asked to rate how they felt “when preparing your speech” and “when you learned that you did not have to give your speech” on a shortened Positive and Negative Affect Schedule (PANAS) yielding total positive affect and negative affect scores [40]. Each of the four measures was found to be reliable (speech preparation positive affect: α = .89; speech preparation negative affect: α = .82; recovery positive affect: α = .88; recovery negative affect: α = .78).

## Results

### Self-report responses to the social evaluative threat task

#### Speech appraisals

We conducted a 2 (depression: present, absent) x 2 (social anxiety: present, absent) x 2 (appraisal type: demands, resources) mixed ANOVA with depression and social anxiety as between-subjects factors and appraisal type as the within-subject factor. This analysis yielded significant interactions of appraisal type and depression, F(1,57) = 7.10, p = .01, and appraisal type and social anxiety, F(1,57) = 17.51, p < .001. In both cases, the presence of a disorder was associated with greater perceived demands than perceived resources (Table 1), indicating that participants with depression and/or social anxiety perceived the social evaluative threat task as threatening.

### Affect

We conducted separate 2 (depression: present, absent) x 2 (social anxiety: present, absent) x 2 (period: speech preparation, recovery) mixed ANOVAs on positive and negative affect, with depression and social anxiety as between-subjects factors and period as a within-subject factor. For positive affect, the ANOVA yielded significant main effects of depression, F(1,57) = 4.14, p = .046, and social anxiety, F(1,57) = 5.12, p = .027. In both cases, the presence of the disorder was associated with lower positive affect than was the absence of the disorder (Table 1). The ANOVA also yielded a significant main effect of period, F(1, 57) = 9.87, p = .003, indicating that participants reported feeling higher positive affect during recovery (Mean = 2.38, SD = .84) than during speech preparation (Mean = 2.03, SD = .85). None of the higher-order interactions was significant. The ANOVA conducted on negative affect yielded significant main effects of social anxiety and period that were qualified by a significant interaction of social anxiety and period, F(1, 57) = 5.56, p = .022. This interaction was due to participants with social anxiety reporting higher levels of negative affect (Mean = 3.40, SD = 1.05) than did participants without social anxiety (Mean = 2.64, SD = 1.06) during speech preparation, F(1,57) = 9.47, p = .003, but not during recovery (social anxiety: Mean = 1.43, SD = .53; no social anxiety: Mean = 1.25, SD = .54; F(1, 57) = .50, p = .48).

### fMRI findings – GLM analysis

#### Overall intercept

**Medial frontal cortex.** On average, participants exhibited increased medial frontal cortex activation during the early and late speech preparation periods (Table 2). **Other regions.** Notably, participants also exhibited decreased activation in a large region of cortex spanning bilateral inferior frontal gyrus and middle cingulate during the instructions period, as well as decreased activation in the anterior cingulate and insula during recovery.

**Table 2:** **GLM analysis of BOLD responses to the social evaluative threat task – overall intercept**^**1**^

**Region**	**x**	**y**	**z**	**voxels**	**vol (mm^3^)**	**Peak t**
Positive Activation						
*Instructions*						
Occipital cortex	4	−64	−8	3772	101844	10.78
Midbrain	8	−8	−10	153	4131	6.37
l. Amygdala	−20	−10	−10	22	594	5.10
r. Middle Temporal G.	56	−34	−2	68	1836	5.37
r. Inferior Frontal G.	44	22	22	142	3834	5.26
l. Inferior Frontal G.	−38	28	20	22	594	4.77
*Early Speech Prep*						
Medial Frontal G./Anterior Cingulate G.	4	22	16	1640	44280	6.61
*Late Speech Prep*						
l. Middle Frontal G.	−32	4	38	24	648	5.85
r. Precentral G.	32	−16	40	21	567	4.08
Medial Frontal G.	2	26	40	14	378	4.30
*Recovery*						
l. Middle Temporal G.	−52	−22	−8	54	1458	5.16
r. Lingual G.	26	−80	−4	15	405	4.48
Midbrain	−2	−28	4	69	1863	5.07
l. Superior Temporal G.	−50	−32	16	140	3780	4.66
l. Middle Frontal G.	−44	28	20	45	1215	4.43
Precuneus	2	−34	44	36	972	4.65
Negative Activation						
*Instructions*						
l. Uncus	−32	2	−28	22	594	−5.03
Bilateral Inferior Frontal G./Middle Cingulate G.	−8	−8	28	6335	171045	−10.32
r. Middle Frontal G.	32	52	8	97	2619	−6.78
r. Middle Frontal G.	−32	52	8	102	2754	−5.53
*Early Speech*						
Occipital Cortex	−2	−64	2	2488	67176	−8.72
l. Middle Frontal G.	−40	22	20	132	3564	−6.18
*Late Speech*						
r. Parahippocampal G.	32	−26	−22	29	783	−4.39
l. Parahippocampal G.	−34	−32	−22	35	945	−4.34
Midbrain	2	−28	2	64	1728	−5.22
*Recovery*						
Pons	−8	−32	−28	69	1863	−4.99
Anterior Cingulate G./Caudate/Insula	8	8	14	2350	63450	−7.05

Note. ^1^ The GLM analysis did not yield any activation differences as a function of diagnosis. l. = left, r. = right.

#### Diagnosis differences

The GLM analysis did not yield any significant main effects of depression and social anxiety, or significant interaction of depression and social anxiety.

### fMRI findings – change-point analysis

#### Overall intercept

**Figure 1:**
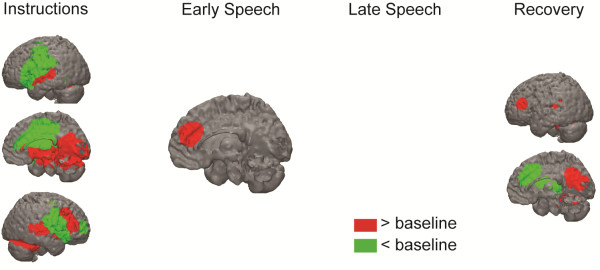
**BOLD responses to social evaluative threat for the overall group intercept.** These BOLD responses represent overall changes in activation (intercept from multiple regression equation) from baseline to the four periods of interest (instructions [60–100 time-points)], early speech preparation [101–130 time-points], late speech preparation [131–160 time-points], and recovery period [161–225 time-points]).

**Medial frontal cortex.** On average, participants exhibited increased medial frontal cortex activation during the early speech preparation period (Figure 1; Table 3). **Other regions.** Notably, participants also exhibited decreased activation in the posterior cingulate during the late speech preparation period and decreased activation in the anterior cingulate during recovery.

**Table 3:** Change-point analysis of the BOLD responses to the social evaluative threat – average (intercept) across all participants

**Region**	**x**	**y**	**Z**	**voxels**	**vol (mm^3^)**	**Peak t**	**Change-point onset**	**Change-point offset**
Activation > Baseline								
*Instructions*								
Occipital cortex	2	−58	−4	3811	102897	18.22	64	106
Midbrain	2	−8	−10	197	5319	7.68	63	78
l. Superior Temporal G.	−52	−4	−2	138	3726	7.89	68	82
r. Superior Temporal G. & Insula	50	−16	4	284	7668	8.44	62	80
r. Inferior Frontal G.	44	28	16	121	3267	7.16	65	82
*Early Speech Prep*								
Medial Frontal G.	4	38	20	73	1971	5.2	108	119
*Late Speech Prep*								
No suprathreshold voxels								
*Recovery*								
r. Fusiform G.	32	−50	−14	98	2646	6.45	164	171
l. Parahippocampal G.	−26	−40	−14	83	2241	7.32	163	171
Precuneus	8	−62	22	200	5400	8.28	163	173
Posterior Cingulate G.	−16	−52	10	28	756	7.05	163	171
l. Superior Temporal G.	−50	−32	16	40	1080	5.04	165	177
l. Middle Frontal G.	−44	34	16	15	405	5.55	164	169
Activation < Baseline								
*Instructions*								
Middle Cingulate G./Bilateral Insula/Parietal Cortex	−8	−8	26	5617	151659	−11.87	63	88
r. Hippocampus	32	−22	−4	27	729	−6.09	66	71
r. Middle Temporal G.	34	52	8	24	648	−4.97	70	80
r. Caudate	22	−32	16	22	594	−5.72	65	71
r. Superior Temporal G.	40	−46	22	19	513	−5.39	63	66
*Early Speech Prep*								
l. Posterior Cingulate G.	−26	−44	34	33	891	−6.19	106	117
*Late Speech Prep*								
No suprathreshold voxels								
*Recovery*								
r. Thalamus	28	−26	−2	35	945	−5.82	165	169
l. Putamen	−32	−22	−2	50	1350	−5.84	165	170
r. Putamen	28	−8	8	78	2106	−6.55	164	171
l. Thalamus	−16	−22	10	25	675	−6.84	165	170
Anterior Cingulate G.	10	26	34	135	3645	−5.58	165	179

*Note.* vol = volume; G. = Gyrus.

#### Depression vs. No Depression

**Figure 2:**
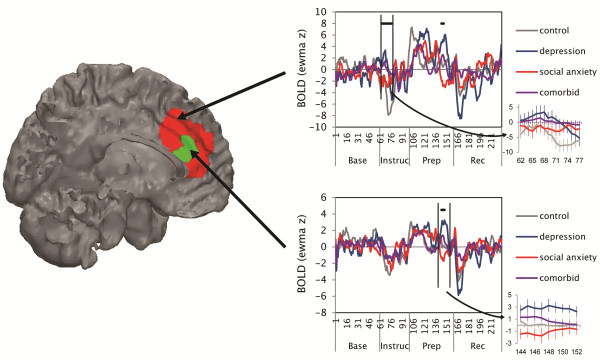
**BOLD responses to social evaluative threat by diagnostic group in the medial frontal cortex.** Each graph plots activation across time: EWMA z = exponentially weighted moving average z-statistic (temporally smoothed deviations from baseline period). Base = baseline, Instruc = instructions period, Prep = speech preparation period, and Rec = recovery period. Solid black bars designate the time-points for which there was a main effect of being diagnosed with depression vs. not having depression; insets show these data with standard error bars. BOLD = blood oxygenation level dependent.

**Medial frontal cortex.** As hypothesized, the change-point analysis yielded a main effect of being diagnosed with depression on medial frontal cortex activation during the late speech preparation period. Whereas participants diagnosed with depression (MDD, MDD/SAD) exhibited a resurgence of medial frontal cortex activation during the late speech preparation period, participants without depression (SAD, CTL) exhibited a return to baseline during this period (Figure 2). There was also a main effect of being diagnosed with depression on medial frontal cortex activation in the instructions period; participants with diagnosed depression did not exhibit the deactivation that was evident in nondepressed participants (Figure 2; Table 4). Notably, these associations between depression and medial frontal cortex activity remained significant when controlling for depression and anxiety symptoms (both ts > 3.8, ps < .001). **Other regions.** As with the medial frontal cortex findings, participants diagnosed with depression exhibited a more extensive pattern of activation than did participants without depression during the instructions (e.g., middle cingulate, precentral gyrus) and late speech preparation (e.g., insula, caudate; Table 4) periods. In the early speech preparation period, participants with depression exhibited greater activation than did participants without depression in the inferior frontal gyrus.

**Table 4:** Change-point analysis of the BOLD responses to the social evaluative threat task of participants with and without major depressive disorder

**Region**	**x**	**y**	**z**	**voxels**	**vol (mm^3^)**	**Peak t**	**change-point onset**	**change-point offset**	**comorbid (~,>,<) pure depression^1^**
Depression > No Depression									
*Instructions*									
r. Insula	38	8	−2	71	1917	7.09	74	82	<
l. Precentral G.*	−50	4	4	54	1458	5.84	99	115	<
l. Precentral G.*	−56	−8	16	15	405	5.83	73	81	<
l. Middle Frontal G.	−40	34	16	14	378	6.88	62	70	~
Medial Frontal G.	4	34	26	88	2376	7.58	62	77	~
l. Inferior Frontal G.	−50	−2	26	19	513	6.79	77	88	
Cingulate G.*	2	4	28	50	1350	6.88	62	81	<
Cingulate G.	−14	−4	26	26	702	5.9	71	81	
Cingulate G.	10	−10	28	24	648	6.11	65	83	<
*Early Speech Prep*									
l. Precentral G.	−56	−4	8	36	972	5.97	100	114	~
l. Prefrontal G.	−50	4	16	20	540	5.54	104	112	~
Cingulate G.	16	−28	38	16	432	6.35	103	111	~
*Late Speech Prep*									
l. Insula	−34	−16	14	14	378	5.56	152	160	
r. Insula	34	−8	22	76	2052	6.01	148	162	
l. Postcentral G.	−52	−22	22	24	648	6.54	157	163	~
r. Caudate	14	2	20	33	891	5.97	151	161	
Cingulate G.	10	34	20	15	405	6.69	144	152	
l. Sub-gyral	−34	−20	34	31	837	5.88	148	162	~
l. Sub-gyral*	−16	−40	34	17	459	6.33	154	161	<
*Recovery*									
r. Cerebellum: Culmen	26	−44	−20	22	594	6.08	164	170	~
Lingual G.*	8	−62	−4	176	4752	7.52	163	171	<
Lingual G.	−8	−82	−2	20	540	6.84	164	169	~
No Depression > Depression									
*Instructions*									
Posterior Cingulate	−4	−44	10	16	432	−6.47	70	74	~
Cuneus	−8	−76	20	18	486	−6.64	69	95	~
r. Cuneus	16	−74	16	34	918	−5.66	85	97	~
*Early Speech Prep*									
l. Caudate	−14	−22	26	16	432	−5.87	116	123	
*Late Speech Prep*									
l. Lentiform Nucleus	−26	8	8	31	837	−6.67	132	142	
*Recovery*									
Posterior Cingulate G.*	14	−56	14	55	1485	−6.53	176	190	>
l. Precentral G.	−44	−10	40	16	432	−5.89	165	173	~

*Note.* These clusters were masked with the clusters that exhibited group average activation differences during the social evaluative threat task. vol = volume; G. = Gyrus. ^1^ Post-hoc group contrasts that test whether comorbid participants exhibited similar (~; not significantly different from each other, but both groups are different than control participants), greater (>), or less (<) activation than participants with pure depression (p < .05). The lack of a symbol means that comorbid participants did not exhibit significantly different activation than control participants and participants with pure depression.

* Those regions for which comorbid participants’ activation significantly differed from both pure depression and pure social anxiety groups.

#### Social Anxiety vs. No Social Anxiety

**Medial frontal cortex.** Contrary to predictions, there was no effect of social anxiety diagnosis on medial frontal cortex activation in the speech preparation period or during recovery. **Other regions.** Instead, participants diagnosed with social anxiety (SAD, MDD/SAD), relative to participants without diagnosed social anxiety (MDD, CTL), exhibited greater activation in the occipital cortex and middle temporal gyrus during instructions as well as less activation in the insula and postcentral gyrus during recovery (Table 5).

**Table 5:** Change-point analysis of the BOLD responses to the social evaluative threat task of participants with and without social anxiety disorder

**Region**	**x**	**y**	**z**	**voxels**	**vol (mm^3^)**	**Peak t**	**change-point onset**	**change-point offset**	**comorbid (~,>,<) social anxiety**
Social Anxiety > No Social Anxiety									
*Instructions*									
Cerebellum: Declive*	8	−58	−14	325	8775	12.52	66	102	<
r. Lingual G.*	26	−74	−2	15	405	5.34	69	79	<
r. Superior Temporal G.	56	−38	4	31	837	7.68	66	83	~
*Early Speech Prep*									
No suprathreshold voxels									
*Late Speech Prep*									
No suprathreshold voxels									
*Recovery*									
r. Cerebellum: Declive*	26	−56	−20	142	3834	9.03	164	174	<
l. Cerebellum: Declive	−20	−62	−20	83	2241	7.45	163	171	~
No Social Anxiety > Social Anxiety									
*Instructions*									
l. Insula	−32	8	−4	54	1458	−7.21	68	80	
r. Precentral G.	50	−8	22	151	4077	−6.6	67	81	~
l. Inferior Frontal G.*	−52	8	10	66	1782	−6.62	68	86	>
r. Middle Frontal G.	34	52	8	19	513	−7.21	68	82	~
Cingulate G.	4	−8	34	237	6399	−7.91	66	91	
l. Cingulate/Supramarginal G.	−26	−26	38	159	4293	−6.94	66	81	~
*Early Speech Prep*									
No suprathreshold voxels									
*Late Speech Prep*									
No suprathreshold voxels									
*Recovery*									
r. Insula	44	−2	−2	103	2781	−6.67	164	184	~
l. Insula	−40	4	2	124	3348	−7.4	164	184	~
l. Postcentral G.	−38	−20	38	29	783	−6.89	166	177	~

*Note.* These clusters were masked with the clusters that exhibited group average activation differences during the social evaluative threat task. vol = volume; G. = Gyrus. ^1^ Post-hoc group contrasts that test whether comorbid participants exhibited similar (~; not significantly different from each other, but both groups are different than control participants), greater (>), or less (<) activation than participants with pure social anxiety (p < .05). The lack of a symbol means that comorbid participants did not exhibit significantly different activation than control participants and participants with pure social anxiety.

* Those regions for which comorbid participants’ activation significantly differed from both pure depression and pure social anxiety groups.

#### Interaction of Depression and Social Anxiety

**Figure 3:**
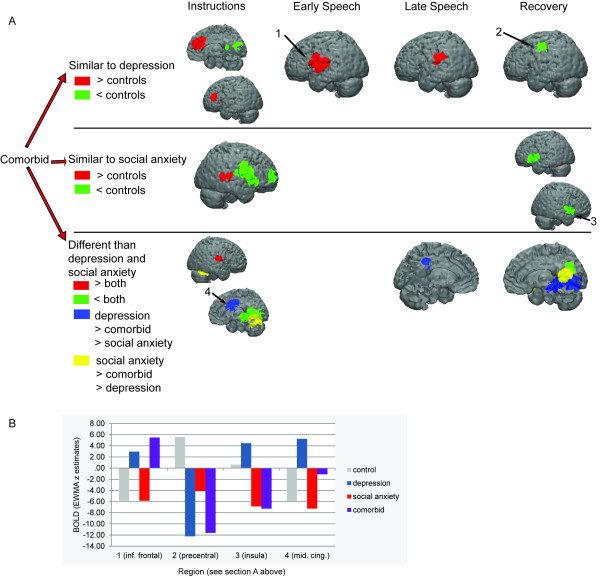
**Post-hoc group contrasts that compare the activation of the comorbid group with that of the pure depression and social anxiety groups. A**. To have demonstrated similar activation as one of the two pure diagnostic groups, the comorbid group must have exhibited activation that was not significantly different from the pure diagnostic group, and the activation of both the comorbid and pure diagnostic groups must have been significantly different than controls. To have demonstrated different activation than the two pure diagnostic groups, the comorbid group must have exhibited significantly different activation from both the pure depression and social anxiety groups. **B**. Each group’s beta estimates of the EWMA z statistic for selected regions. BOLD = blood oxygenation level dependent. EWMA z = exponentially weighted moving average z-statistic (temporally smoothed deviations from baseline period).

**Medial frontal cortex.** There was no interaction of diagnoses of depression and social anxiety on activation in the medial frontal cortex. **Other regions.** Participants diagnosed with comorbid depression/social anxiety exhibited different patterns of activation than did participants with non-comorbid diagnoses in several regions (Figure 3; Table 6). The comorbid participants exhibited intermediate activation in the middle cingulate cortex and precentral gyrus (less than the pure depression group and more than the pure social anxiety group; Figure 3) as well as the posterior cingulate (less than the pure social anxiety group and more than the pure depression group). The comorbid participants also exhibited activation similar to that exhibited by the control participants in several regions, including greater activation than participants with pure depression or pure social anxiety in the insula (instructions) and middle temporal gyrus (recovery), and less activation than the pure diagnostic groups in the cerebellum (instructions) and cuneus (instructions/recovery).

**Table 6:** Change-point analysis of the interaction of depression and social anxiety in BOLD responses to the social evaluative threat task

**Region**	**x**	**y**	**z**	**voxels**	**vol (mm^3^)**	**Peak t**	**change-point onset**	**change-point offset**	**Control**	**Depression**	**Social Anxiety**	**Co-morbid**
											Comorbid and/or Control > Depression and/or Social Anxiety												
*Instructions*													
l. Parahippocampal G	−40	−38	−2	34	918	7.3	65	82	2.52^b^	−6.09^a^	−8.17^a^	−1.43^ab^	
l. Insula	−40	4	−2	28	756	6.84	64	69	7.10^c^	−8.56^a^	−5.08^ab^	−0.16^b^	
l. Insula	−38	−16	8	15	405	5.77	70	78	3.26^b^	−6.15^a^	−6.46^a^	−2.08^ab^	
l. Insula	−40	−8	20	195	5265	8.47	64	79	2.25^b^	−9.35^a^	−9.24^a^	−4.41^a^	
r. Insula*	50	−14	14	30	810	5.78	65	71	2.56^b^	−7.62^a^	−7.50^a^	1.00^b^	
l. Precentral G.	−38	8	22	15	405	5.99	65	75	0.03^b^	−4.14^ab^	−6.87^a^	0.26^b^	
*Early Speech Prep*													
l. Insula	−50	−34	22	36	972	5.89	115	121	9.46^c^	−11.72^a^	−1.47^b^	2.00^b^	
*Late Speech Prep*													
l. Lentiform Nucleus	−32	4	−2	15	405	5.58	148	157	3.51^b^	−3.26^a^	−3.48^a^	1.39^ab^	
*Recovery*													
l. Middle Temporal G.*	−44	−38	2	36	972	7.3	160	170	4.01^b^	−7.12^a^	−6.40^a^	2.08^b^	
l. Precentral G.	−38	−14	32	23	621	7.07	164	177	4.36^b^	−8.80^a^	−4.96^a^	−3.08^a^	
Depression and/or Social Anxiety > Comorbid and/or Control													
*Instructions*													
Cerebellum: Declive*	8	−62	−20	14	378	−5.38	90	95	−7.42^a^	10.43^c^	26.22^d^	2.94^b^	
Cuneus*	−8	−68	4	446	12042	−7.94	65	84	3.28^a^	27.79^c^	29.39^c^	12.44^b^	
r. Inferior Frontal G.	40	20	−10	15	405	−6.07	69	81	−11.20^a^	7.13^c^	−4.78^b^	−8.58^ab^	
r. Insula	40	4	−4	23	621	−6.57	71	84	−11.09^a^	8.04^b^	−7.03^a^	−7.09^a^	
r. Cuneus*	22	−76	8	37	999	−6.06	67	79	−1.70^a^	15.35^b^	21.56^c^	3.66^a^	
*Early Speech Prep*													
No suprathreshold voxels													
*Late Speech Prep*													
No suprathreshold voxels													
*Recovery*													
Lingual G.	8	−56	−4	448	12096	−7.4	161	172	−3.95^a^	27.24^c^	18.43^bc^	16.44^b^	
Precuneus*	−2	−68	26	21	567	−6.21	164	171	−6.24^a^	24.31^c^	23.38^c^	12.11^b^	

*Note.* These clusters were masked with the clusters that exhibited group average activation differences during the social evaluative threat task. vol = volume; G. = Gyrus. Different subscripts represent significantly differences between the groups, calculated using simple slopes analyses on the beta coefficients (presented here) from the multiple regression models.

* Those regions for which comorbid participants’ activation significantly differed from both pure depression and pure social anxiety groups.

### Correlations among brain activation and self-reported responses to the social evaluative threat task

#### Regions associated with depression

We examined whether the differential medial frontal cortex responses (MDD > No MDD) during the instructions and late speech preparation periods were related to self-reported responses to the task (α_corr_ = .05/6 = .008). No correlations survived Bonferroni correction.

### Regions associated with social anxiety

We next examined the correlations between self-reported responses to the speech task and differential responses in the lingual gyrus and superior temporal gyrus during the instructions period (SAD > No SAD) and in the insula during the recovery period (SAD < No SAD) – regions that may represent the hypothesized ‘vigilance-avoidance’ pattern of responding (α_corr_ = .05/6 = .008). Across all participants, no correlations survived Bonferroni correction. Across only the participants with social anxiety, activation in the lingual gyrus during the instructions period was negatively correlated with negative affect to preparing the speech, r(32) = −.49, p = .004, and perceived demands of the speech, r(32) = −.47, p = .006. In addition, left insula activation during the recovery period was positively correlated with perceived demands of the speech, r(32) = .48, p = .005.

## Discussion

The present study was designed to examine magnitude and timing of neural responses to stress in individuals diagnosed with non-comorbid depression, non-comorbid social anxiety, and comorbid depression/social anxiety. All groups exhibited increased medial frontal cortex activation during the early speech preparation, which is consistent with findings from previous studies [26], followed by decreased activation toward the middle of the speech anticipation period. As we predicted, the change-point analysis revealed that participants with depression (both pure and comorbid) exhibited greater medial frontal cortex activation than those without depression late in the stress task. Notably, whereas we had initially hypothesized that depressed participants would exhibit a sustained pattern of activation, we found instead that depressed participants exhibited a resurgence of medial frontal cortex activation late in the stress task. This late resurgence of medial frontal cortex activation suggests that participants with depression re-engaged with the stressor, although it is unclear what psychological functions subserved this re-engagement. This medial frontal cortex activation during the late speech preparation period was not significantly correlated with any self-reported responses to the speech task, suggesting that depressed participants’ re-engagement with the stressor was not due to a more intense negative emotional response to the stressor. One possible explanation for this finding is that this late activation in the medial prefrontal cortex for the depressed participants was due to rumination about negative aspects of the stress task. Indeed, activation in the medial frontal cortex has been found to be associated with functions that subserve rumination about negative events: self-referential processing, [41] self-conscious emotional reactivity, [42] and extended duration of negative emotions [43]. Alternatively, this region has also been found to be associated with generating the arousal necessary for anticipated effort, [26,44] suggesting the possibility that this pattern of activation was due to depressed participants re-exerting the effort they anticipated it would take to perform the upcoming speech. Future investigations that explicitly assess ruminative thoughts as well as motivation/anticipated effort in depression are necessary to test the veracity of these formulations.

The vigilance-avoidance hypothesis for participants with social anxiety was only partially supported. Participants without depression (i.e., those with either social anxiety or no disorder) exhibited decreased activation in the medial frontal cortex during the instructions period. Consistent with the vigilance hypothesis, deactivation in this region co-occurs with cognitive engagement to external stimuli, [45] suggesting that participants with pure social anxiety were paying relatively more attention to external stimuli (the speech instructions) than were participants with depression. Further supporting this formulation, participants with both pure social anxiety and comorbid social anxiety/depression exhibited greater activation in other regions associated with attending to external stimuli, including the occipital cortex and superior temporal gyrus [46], than did participants without social anxiety. Consistent with the avoidance hypothesis, during recovery, social anxiety and comorbid participants exhibited decreased activation in the insula, a region associated with monitoring internal states [47], especially those associated with threat [48]. Together, this pattern of findings suggest that participants with social anxiety exhibited heightened engagement with the stressor when first learning about it (during instructions period) and significant disengagement from the stressor when learning that they would not have to give a speech after all. In addition, we found evidence that this vigilance-avoidance pattern of brain activation may have been adaptive. Across socially anxious participants, the tendency to exhibit greater lingual gyrus activation during the instructions period and decreased insula activation during recovery predicted participants’ tendency to perceive the speech task as less demanding. Contrary to predictions, however, having social anxiety did not predict greater medial frontal cortex activation during the early part of the stressor. One possibility that should be tested in future investigations is that the preparation of the speech was too threatening for participants. Indeed, individual differences in social anxiety have been found to be better predictors of differential responses to mildly threatening social situations than to highly threatening social situations [49].

As predicted, the comorbid participants responded similarly to the participants with pure depression and pure social anxiety in a majority of the suprathreshold voxels. Notably, however, during the speech period, the comorbid participants exhibited activation patterns that more closely matched the participants with pure depression than the participants with pure social anxiety (Figure 3). In future studies, investigators should examine whether this asymmetrical influence of these two diagnoses on brain responses to social threat extend to other facets of neural and behavioral functioning. In the few brain regions in which the comorbid participants exhibited different activations than did the participants with pure diagnoses, two patterns emerged. First, the comorbid participants exhibited intermediate activation levels in the middle cingulate cortex, precentral gyrus, and posterior cingulate. This finding raises the intriguing possibility that in these regions the psychological and neural effects of having both depression and social anxiety interact. Second, compared with the pure diagnostic groups, the comorbid group and never-disordered control participants exhibited more tempered responses in the insula (less deactivation) and cuneus (less activation). Future investigations should target these regions as possible sites of dysregulation for individuals with comorbid depression and social anxiety in which neural reactivity to stress is dampened despite heightened self-reported reactivity.

In addition to unpacking the psychological mechanisms that underlie these differences in activation among the diagnostic groups, future studies that replicate and extend this work should address other elements of the current design. For example, investigators should recruit larger samples of both male and female medication-free participants to replicate and extend the present findings to determine whether they generalize to males and to ensure that they are not influenced by medication status. Also, although the speech stressor we used induced similar levels of neural activation during the early-stress period in the depressed and socially anxious participants, the social threat inherent in this task is theoretically more relevant to social anxiety than to depression. Indeed, the participants with social anxiety did report a slightly higher increase in negative affect in response to the social threat stressor than did the participants with depression. By exploring more depression-relevant stressors, future studies should be able to distinguish activation patterns that generalize to other types of stressors from patterns that are specific to the speech stressor.

## Conclusions

In sum, the present study is among the first to delineate the neural functioning of comorbidity, and is the first to examine specifically the neural functioning of comorbid depression and social anxiety. We used innovative statistical and methodological techniques to probe temporal aspects of stress-induced neural responding and demonstrated that change-point analyses are more sensitive to temporal differences in activation than standard GLM analyses. This change-point approach allowed us to identify both when and where neural activation differences occurred as a function of diagnosis and yielded several intriguing patterns of activation. Future investigations should assess more explicitly the psychological mechanisms that might underlie these neural activation patterns, as well as possible diagnosis-related differences in functional connectivity among these regions [50] during exposure to stress. Elucidating these unique and shared neural responses to social evaluative threat in individuals with pure depression, pure social anxiety, and comorbid depression/social anxiety may facilitate the selection or development of effective interventions for these debilitating disorders.

## Competing interests

The authors declare that, except for income received from our primary employer, no financial support or compensation has been received from any individual or corporate entity over the past three years for research or professional service and there are no personal financial holdings that could be perceived as constituting a potential conflict of interest.

## Authors’ contributions

CW helped conceive of the design of the study, conducted the data analyses, and took the lead on writing the manuscript. JPH and MCC helped conceive of the design of the study, conducted the fMRI sessions, consulted on data analyses, and contributed to writing the manuscript. JJ helped conceive of the design of the study and contributed to writing the manuscript. IHG helped conceive of the design of the study, consulted on data analyses, and contributed to writing the manuscript. All authors have read and approve of this manuscript.

## Supplementary Material

Additional file 1 Supplementary information and tables. Click here for file
