# Supplementary material for: Neural temporal dynamics of stress in comorbid major depressive disorder and social anxiety disorder
Source: Biol Mood Anxiety Disord. 2012 Jun 22;2:11. doi: 10.1186/2045-5380-2-11 (PMC3583464; doi:10.1186/2045-5380-2-11)
Supplement: Additional file 1 — Supplementary information and tables. [file 2045-5380-2-11-S1.docx]

Supplementary information and tables.

**Medication**

Because several of the participants in the three clinical groups were taking psychotropic medications at the time of the scan, we accounted for the potential effects of medication status on functional neuroimaging data by including continuous medication covariates in our random effects analysis. We created two dosage response covariates, one reflecting antidepressant load and another reflecting anxiolytic/sedative load (Sackheim, 2001). These values were summed in those cases in which an individual was taking more than one antidepressant or anxiolytic/sedative. Importantly, our main findings in the medial prefrontal cortex remain unchanged when only including medication-free participants in the analyses.

**Table 1**

List of medications taken by group

| CTL (0/17) | MDD (8/12) | SAD (3/17) | MDD/SAD (5/16) |
| --- | --- | --- | --- |
| None | Ambien | Citalopram | Celexa |
|  | Buproprion | Lorazepam | Clonazepam |
|  | BuSpar | Remeron | Cymbalta |
|  | Effexor | Trazadone | Lexapro |
|  | Lexapro | Valium | Mirtazapine |
|  | Paxil |  | Prozac |
|  | Pristiq |  | Topamax |
|  | Prozac |  | Wellbutrin |
|  | Suboxone |  | Xanax |
|  | Temazepam |  |  |
|  | Trazadone |  |  |
|  | Wellbutrin |  |  |
|  | Zolpidem |  |  |

* Note. Numbers of participants on medication within each group are in parentheses.

**Table 2**

Demographic characteristics, clinical characteristics, and self-reported responses to the social evaluative threat task of the diagnosed groups

|  | Depression (n = 12) | Social Anxiety (n = 17) | Comorbid (n = 17) | Control (n =16) | Interaction of depression and social anxiety | Main effect of having depression | Main effect of having social anxiety |
| --- | --- | --- | --- | --- | --- | --- | --- |
| G^2^ | | | | | | | |
| Race (% Caucasian) | 75% | 71% | 76% | 50% | 5.10 | 2.34 | 1.84 |
| Current treatment | 33% | 13% | 35% | 0% | 9.82* | 7.22* | .60 |
| F | | | | | | | |
| Age | 39.67 (11.30) | 32.82 (12.34) | 29.88 (10.92) | 33.64 (9.36) | 2.40 | .28 | 3.36 |
| Income^ab^ | 3.70 (2.11) | 4.08 (1.44) | 2.60 (1.55) | 4.00 (1.75) | 1.52 | 3.47 | 1.15 |
| Education (years)^c^ | 15.80 (2.69) | 16.06 (1.12) | 14.92 (2.25) | 17.80 (2.73) | .48 | 6.27* | 4.32* |
| Depression (BDI-II) | 26.58 (13.85) | 11.65 (8.82) | 30.35 (10.83) | 2.06 (3.15) | .63 | 100.85* | 13.88* |
| Social Anxiety (SPAI) | 63.31 (29.67) | 91.47 (26.11) | 98.55 (17.81) | 29.79 (28.88) | 3.70 | 8.73* | 49.71* |
| Anti-Depressant Load | 1.75 (2.01) | .41 (1.18) | 1.12 (2.12) | .00 (.00) | 1.74 | 9.61* | .08 |
| Anti-Anxiety Load | .33 (.65) | .12 (.33) | .18 (.39) | .00 (.00) | 1.86 | 3.80* | .04 |
| Positive affect during speech prep | 1.91 (.98) | 1.92 (.67) | 1.74 (.83) | 2.62 (.92) | .65 | 3.99 | 5.54* |
| Negative affect during speech prep | 3.02 (1.16) | 3.33 (1.06) | 3.45 (1.04) | 2.22 (.81) | 1.15 | 2.89 | 7.86* |
| Positive affect after speech prep | 2.27 (.85) | 2.35 (.88) | 2.13 (.59) | 2.74 (1.01) | .44 | 2.20 | 2.30 |
| Negative affect after speech prep | 1.20 (.45) | 1.34 (.42) | 1.53 (.64) | 1.28 (.56) | .93 | .20 | 1.60 |

BDI-II = Beck Depression Inventory; SPAI = Social Phobia and Anxiety Inventory; PA = positive affect; NA = negative affect. Standard deviations are in parentheses. * p < .05.

^a^ Income was measured on an ordinal scale (1 = less than 10k, 2 = 10-25k, 3 = 25-50k, 4 = 50-75k, 5 = 75-100k, 6 = more than 100k).

^b^ N = 52 (Depression, n = 10; Social Anxiety, n = 13; Comorbid, n = 15; Control, n = 14)

^c^ N = 50 (Depression, n = 11; Social Anxiety, n = 16; Comorbid, n = 13; Control, n = 10)

**Table 3**

BOLD responses to the social evaluative threat – average (intercept) across all participants

| Region | x | y | Z | voxels | vol (mm^3^) | Peak z | Change-point onset | Change-point offset |
| --- | --- | --- | --- | --- | --- | --- | --- | --- |
| Activation > Baseline | | | | | | | | |
| *Instructions* |  |  |  |  |  |  |  |  |
| Occipital cortex | 2 | -58 | -4 | 3811 | 102897 | 18.22 | 64 | 106 |
| Midbrain | 2 | -8 | -10 | 197 | 5319 | 7.68 | 63 | 78 |
| Superior Temporal G. | -52 | -4 | -2 | 138 | 3726 | 7.89 | 68 | 82 |
| Superior Temporal G. & Insula | 50 | -16 | 4 | 284 | 7668 | 8.44 | 62 | 80 |
| Inferior Frontal G. | 44 | 28 | 16 | 121 | 3267 | 7.16 | 65 | 82 |
| *Early Speech Prep* |  |  |  |  |  |  |  |  |
| Medial Frontal G. | 4 | 38 | 20 | 73 | 1971 | 5.2 | 108 | 119 |
| *Late Speech Prep* |  |  |  |  |  |  |  |  |
| No suprathreshold voxels |  |  |  |  |  |  |  |  |
| *Recovery* |  |  |  |  |  |  |  |  |
| Fusiform G. | 32 | -50 | -14 | 98 | 2646 | 6.45 | 164 | 171 |
| Parahippocampal G. | -26 | -40 | -14 | 83 | 2241 | 7.32 | 163 | 171 |
| Precuneus | 8 | -62 | 22 | 200 | 5400 | 8.28 | 163 | 173 |
| Posterior Cingulate G. | -16 | -52 | 10 | 28 | 756 | 7.05 | 163 | 171 |
| Superior Temporal G. | -50 | -32 | 16 | 40 | 1080 | 5.04 | 165 | 177 |
| Middle Frontal G. | -44 | 34 | 16 | 15 | 405 | 5.55 | 164 | 169 |
| Activation < Baseline | | | | | | | | |
| *Instructions* |  |  |  |  |  |  |  |  |
| No suprathreshold voxels |  |  |  |  |  |  |  |  |
| *Early Speech Prep* |  |  |  |  |  |  |  |  |
| Middle Cingulate G./Bilateral Insula/Parietal Cortex | -8 | -8 | 26 | 5617 | 151659 | -11.87 | 63 | 88 |
| Hippocampus | 32 | -22 | -4 | 27 | 729 | -6.09 | 66 | 71 |
| Middle Temporal G. | 34 | 52 | 8 | 24 | 648 | -4.97 | 70 | 80 |
| Caudate | 22 | -32 | 16 | 22 | 594 | -5.72 | 65 | 71 |
| Superior Temporal G. | 40 | -46 | 22 | 19 | 513 | -5.39 | 63 | 66 |
| *Late Speech Prep* |  |  |  |  |  |  |  |  |
| Posterior Cingulate G. | -26 | -44 | 34 | 33 | 891 | -6.19 | 106 | 117 |
| *Recovery* |  |  |  |  |  |  |  |  |
| Thalamus | 28 | -26 | -2 | 35 | 945 | -5.82 | 165 | 169 |
| Putamen | -32 | -22 | -2 | 50 | 1350 | -5.84 | 165 | 170 |
| Putamen | 28 | -8 | 8 | 78 | 2106 | -6.55 | 164 | 171 |
| Thalamus | -16 | -22 | 10 | 25 | 675 | -6.84 | 165 | 170 |
| Anterior Cingulate G. | 10 | 26 | 34 | 135 | 3645 | -5.58 | 165 | 179 |

*Note.* vol = volume; G. = Gyrus.
